# Supplementary material for: Estimating total human deaths from rabies: A statistical framework leveraging reports of rabies transmission from deceased organ and tissue donors
Source: PLoS Negl Trop Dis. 2026 Sep 21;20(9):e0014731. doi: 10.1371/journal.pntd.0014731 (PMC13592719; doi:10.1371/journal.pntd.0014731)
Supplement: Supplementary file 6 [file pntd.0014731.s005.zip › Result_A/Sri_Lanka/Figure_S3_Sri_Lanka.pdf]

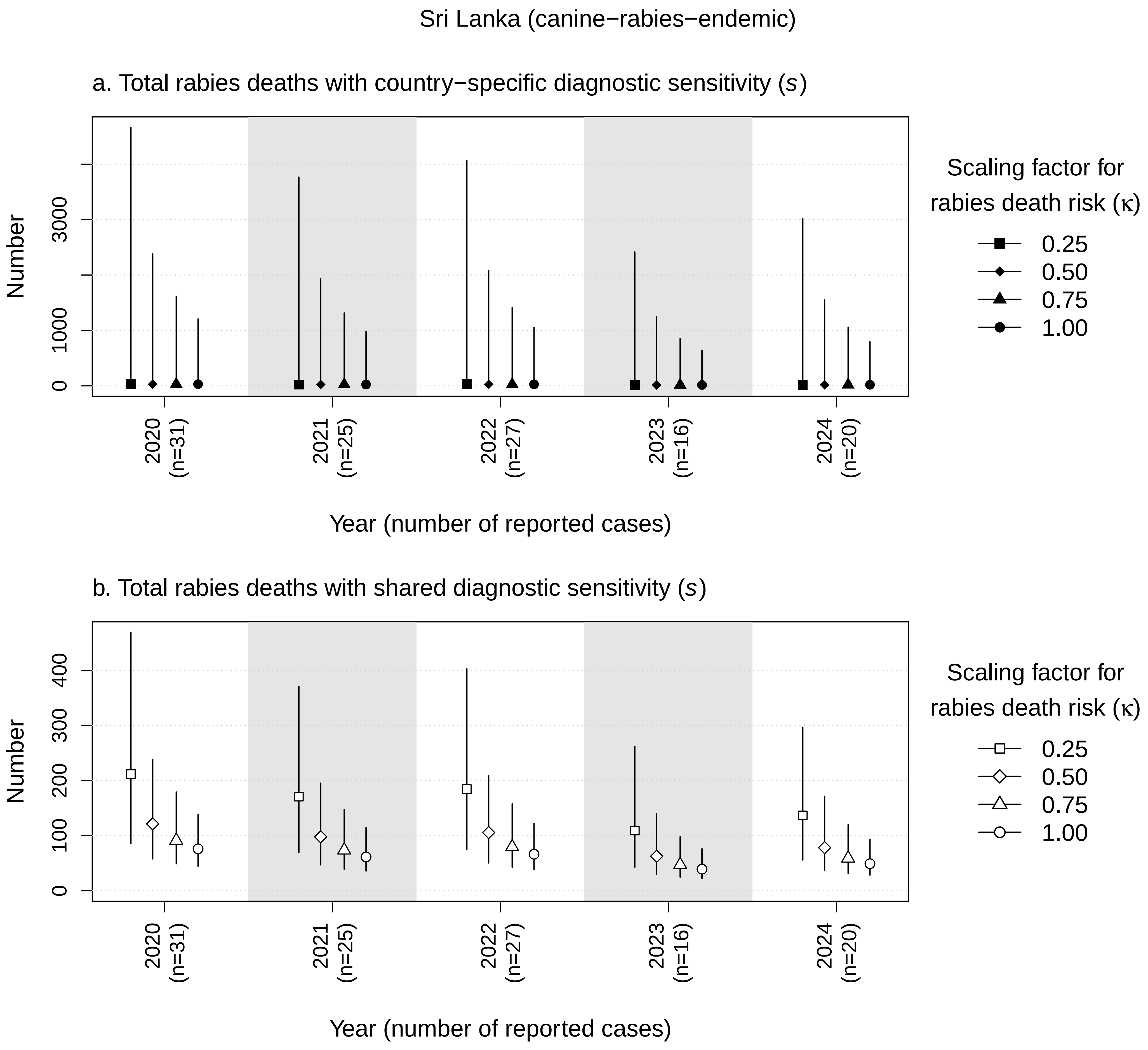

Sri Lanka (canine−rabies−endemic)
a. Total rabies deaths with country−specific diagnostic sensitivity (s)
Number
0
1000
3000
2020 (n=31)
2021 (n=25)
2022 (n=27)
2023 (n=16)
2024 (n=20)
Year (number of reported cases)
Scaling factor for rabies death risk (κ)
0.25
0.50
0.75
1.00
b. Total rabies deaths with shared diagnostic sensitivity (s)
Number
0
100
200
300
400
2020 (n=31)
2021 (n=25)
2022 (n=27)
2023 (n=16)
2024 (n=20)
Year (number of reported cases)
Scaling factor for rabies death risk (κ)
0.25
0.50
0.75
1.00
